# Supplementary material for: The Availability of Essential Antimicrobials in Public and Private Sector Facilities: A Cross-Sectional Survey in a District of North India
Source: Antibiotics (Basel). 2024 Jan 29;13(2):131. doi: 10.3390/antibiotics13020131 (PMC10886159; doi:10.3390/antibiotics13020131)
Supplement: Supplementary file 1 [file antibiotics-13-00131-s001.zip › antibiotics-2765598-Table S1-S4.pdf]

Table S1. Selected (secondary) list of antibiotics for survey

S. No.	ANTIBIOTICS	Formulation	Strength	Healthcare facility level
1.	Amoxicillin	Capsule	250 mg, 500 mg	P,S,T
		Suspension	125 mg/ 5 mL	P,S,T
2.	Amoxicillin + clavulanic acid	Tablet	500/125 mg	P,S,T
		Injection	0.6 g, 1.2 g	S,T
		Suspension	228.5 mg/5 mL	P,S,T
3.	Cefazolin	Injection	500 mg, 1 g	P,S,T
4.	Cefuroxime	Injection	750 mg/ 1.5 g	S,T
5.	Cefixime	Tablet	100, 200, 400 mg	S,T
		Syrup	50 mg/ 5 mL	S,T
6.	Ceftriaxone	Injection	250 mg, 500 mg, 1 g	S,T
7.	Azithromycin	Tablet	250 mg, 500 mg	P,S,T
		Injection	500 mg	S,T
		Suspension	200 mg/ 5 mL	P,S,T
8.	Ciprofloxacin	Tablet	250 mg, 500 mg	P,S,T
		Injection	200 mg/100 mL	P,S,T
9.	Levofloxacin	Tablet	500 mg	S,T
10.	Norfloxacin	Tablet	400 mg	P,S,T
11.	Ofloxacin	Tablet	100, 200, 400 mg	S,T
12.	Cotrimoxazole	Tablet	400/80, 800/160 mg	P,S,T
		Suspension	200+40 mg/ 5 mL	P,S,T
13.	Doxycycline	Capsule	100 mg	P,S,T
14.	Amikacin	Injection	100, 250, 500 mg/2 mL	P,S,T
15.	Metronidazole	Tablet	200 mg, 400 mg	P,S,T
		Injection	500 mg/ 100 mL	P,S,T
		Suspension	200 mg/ 5 mL	P,S,T
16.	Vancomycin	Injection	250 mg, 500 mg, 1 g	T
17.	Nitrofurantoin	Tablet	100 mg	P,S,T
18.	Linezolid	Tablet	600 mg	T
		Infusion	2 mg/mL 100, 300mL	T
19.	Meropenem	Injection	250 mg, 500 mg, 1 g	T

Table S2. Selected (secondary) list of other antimicrobials for survey

S. No.	Name of antimicrobial	Formulation	Strength	Healthcare facility level
**ANTHELMINTHICS**				
1.	Albendazole	Tablet	400 mg	P,S,T
		Suspension	200 mg/ 5 mL	P,S,T
2.	Ivermectin	Tablet	6 mg	P,S,T
**ANTIVIRALS**				
3.	Acyclovir	Tablet	200 mg, 400 mg	P,S,T
		Injection	250 mg, 500 mg	S,T
		Suspension	400 mg/ 5 mL	T
4.	Oseltamivir	Tablet	75 mg	P,S,T
		Syrup	6 mg/ mL, 60 mL	P,S,T
**ANTIFUNGALS**				
5.	Liposomal Amphotericin B	Injection	50 mg/ vial	S, T
6.	Clotrimazole	Pessary	100 mg, 200 mg	P,S,T
7.	Fluconazole	Tablet	100, 150, 200, 400 mg	P,S,T
		Injection	2 mg/ mL	T
8.	Itraconazole	Tablet	200 mg	P,S,T
9.	Voriconazole	Tablet	200 mg	T
		Injection	200 mg	T
**ANTIPROTOZOALS**				
10.	Tinidazole	Tablet	500 mg	P,S,T

Table S3. List of anti-infective medicines included in NLEM 2015.

5.7	Phenobarbitone	P,S,T	Tablet 30 mgTablet 60 mgOral liquid 20 mg/5 ml
		S,T	Injection 200 mg/ml
5.8	Phenytoin	P,S,T	Tablet 50 mg<br>Tablet 100 mg<br>Tablet 300 mg<br>ER Tablet 300 mg<br>Oral liquid 30 mg/5 ml<br>Oral liquid 125 mg/5 ml<br>Injection 25 mg/ml<br>Injection 50 mg/ml
5.9	Sodium valproate	P,S,T	Tablet 200 mg<br>Tablet 300 mg<br>CR Tablet 300 mg<br>Tablet 500 mg<br>CR Tablet 500 mg<br>Oral liquid 200 mg/5ml
		T	Injection 100 mg/ml
**Section 6–Anti infective medicines**			
**6.1–Anthelminthics**			
**6.1.1–Intestinal anthelminthics**			
	**Medicine**	**Level of Healthcare**	**Dosage form and strength**
6.1.1.1	Albendazole	P,S,T	Tablet 400 mg<br>Oral liquid 200 mg/5 ml

6.1.1.2	Mebendazole	P,S,T	Tablet 100 mg<br>Oral liquid 100 mg/5 ml
**6.1.2–Antifilarial**			
	**Medicine**	**Level of Healthcare**	**Dosage form and strength**
6.1.2.1	Diethylcarbamazin e	P,S,T	Tablet 50 mg<br>Tablet 100 mg<br>Oral liquid 120 mg/5 ml
**6.1.3–Anti-schistosomal & anti-trematodal medicine**			
	**Medicine**	**Level of Healthcare**	**Dosage form and strength**
6.1.3.1	Praziquantel	S,T	Tablet 600 mg
**6.2–Antibacterials**			
**6.2.1–Beta lactam medicines**			
	**Medicine**	**Level of Healthcare**	**Dosage form and strength**
6.2.1.1	Amoxicillin	P,S,T	Capsule 250 mg<br>Capsule 500 mg<br>Oral liquid 250 mg/5 ml

6.2.1.2	Amoxicillin (A) + Clavulanic acid (B)	P,S,T	Tablet 500 mg (A) + 125 mg (B)<br>Oral liquid 200 mg (A) + 28.5 mg (B)/5 ml<br>Dry Syrup 125 mg (A) + 31.25 (B)/5 ml
		S,T	Powder for Injection 500 mg (A) + 100 mg (B)<br>Powder for Injection 1 g (A) + 200 mg (B)
6.2.1.3	Ampicillin	P,S,T	Powder for Injection 500 mg<br>Powder for Injection 1 g
6.2.1.4	Benzathine benzylpenicillin	P,S,T	Powder for Injection 6 lac units<br>Powder for Injection 12 lac units
6.2.1.5	Benzyl penicillin	P,S,T	Powder for Injection 10 lac units
6.2.1.6	Cefadroxil	P,S,T	Tablet 500 mg<br>Tablet 1 g<br>Oral liquid 125 mg/5 ml
6.2.1.7	Cefazolin	P,S,T	Powder for Injection 500 mg<br>Powder for Injection 1 g

6.2.1.8	Cefixime	S,T	Tablet 200 mg<br>Tablet 400 mg<br>Oral liquid 50 mg/5 ml<br>Oral liquid 100 mg/5 ml
6.2.1.9	Cefotaxime	S,T	Powder for Injection 250 mg<br>Powder for Injection 500 mg<br>Powder for Injection 1 g
6.2.1.10	Ceftazidime	S,T	Powder for Injection 250 mg<br>Powder for Injection 1 g
6.2.1.11	Ceftriaxone	S,T	Powder for Injection 250 mg<br>Powder for Injection 500 mg<br>Powder for Injection 1 g<br>Powder for Injection 2 g
6.2.1.12	Cloxacillin	P,S,T	Capsule 250 mg<br>Capsule 500 mg<br>Oral Liquid 125 mg/5 ml<br>Powder for Injection 250 mg
6.2.1.13	Piperacillin (A) + Tazobactam (B)	T	Powder for Injection 1 g (A) + 125 mg (B)<br>Powder for Injection 2 g (A) + 250 mg (B)<br>Powder for Injection 4 g (A) + 500 mg (B)

6.2.2–Other antibacterials			
	**Medicine**	**Level of Healthcare**	**Dosage form and strength**
6.2.2.1	Azithromycin	P,S,T	Tablet 250 mg<br>Tablet 500 mg<br>Oral liquid 200 mg/5ml<br>Powder for Injection 500 mg
6.2.2.2	Ciprofloxacin	P,S,T	Tablet 250 mg<br>Tablet 500 mg<br>Oral liquid 250 mg/5ml<br>Injection 200 mg/100 ml
6.2.2.3	Clarithromycin	S,T	Tablet 250 mg<br>Tablet 500 mg<br>Oral liquid 125mg/5 ml
6.2.2.4	Co-trimoxazole [Sulphamethoxazole (A) + Trimethoprim (B)]	P,S,T	Tablet 400 mg (A) + 80 mg (B)<br>Tablet 800 mg (A) + 160 mg (B)<br>Oral liquid 200 mg (A) + 40 mg (B)/5 ml
6.2.2.5	Doxycycline	P,S,T	Capsule 100 mg<br>Dry Syrup 50mg/5 ml
6.2.2.6	Gentamicin	P,S,T	Injection 10 mg/ml<br>Injection 40 mg/ml
6.2.2.7	Metronidazole	P,S,T	Tablet 200 mg<br>Tablet 400 mg<br>Oral liquid 200 mg/5 ml<br>Injection 500 mg/100 ml
6.2.2.8	Nitrofurantoin	P,S,T	Tablet 100 mg<br>Oral liquid 25 mg/5 ml

6.2.2.9	Vancomycin	T	Powder for Injection 250 mg<br>Powder for Injection 500 mg<br>Powder for Injection 1 g
**6.2.3–Antileprosy medicines**			
	**Medicine**	**Level of Healthcare**	**Dosage form and strength**
6.2.3.1	Clofazimine	P,S,T	Capsule 50 mg<br>Capsule 100 mg
6.2.3.2	Dapsone	P,S,T	Tablet 25 mg<br>Tablet 50 mg<br>Tablet 100 mg
6.2.3.3	Rifampicin	P,S,T	Capsule 150 mg<br>Capsule 300 mg
**6.2.4–Antituberculosis medicines**			
	**Medicine**	**Level of Healthcare**	**Dosage form and strength**
6.2.4.1	Capreomycin	P, S, T	Powder for Injection 1 g
6.2.4.2	Cycloserine	P, S, T	Capsule 125 mg<br>Capsule 250 mg
6.2.4.3	Ethambutol	P,S,T	Tablet 200 mg<br>Tablet 400 mg<br>Tablet 600 mg<br>Tablet 800 mg
6.2.4.4	Ethionamide	P, S, T	Tablet 125 mg<br>Tablet 250 mg

6.2.4.1 3	Rifampicin	P,S,T	Capsule 150 mg<br>Capsule 300 mg<br>Capsule 450 mg<br>Capsule 600 mg<br>Oral liquid 100 mg/5 ml
6.2.4.1 4	Streptomycin	P,S,T	Powder for Injection 750 mg<br>Powder for Injection 1 g
**6.3–Antifungal medicines**			
	**Medicine**	**Level of Healthcare**	**Dosage form and strength**
6.3.1	Amphotericin B<br>a) Amphotericin B (conventional)<br>b) Lipid/ Liposomal Amphotericin B	S,T	Powder for Injection 50 mg
6.3.2	Clotrimazole	P,S,T	Pessary 100 mg
6.3.3	Fluconazole	P,S,T	Tablet 100 mgTablet 150 mgTablet 200 mgTablet 400 mgOral liquid 50 mg/5 ml
		S,T	Injection 200 mg /100 ml
6.3.4	Griseofulvin	P,S,T	Tablet 125 mg<br>Tablet 250 mg<br>Tablet 375 mg

6.3.5	Nystatin	P,S,T	Tablet 500,000 IU<br>Pessary 100,000 IU<br>Oral Liquid 100, 000 IU/ml
**6.4–Antiviral medicines**			
**6.4.1–Antiherpes medicines**			
	**Medicine**	**Level of Healthcare**	**Dosage form and strength**
6.4.1.1	Acyclovir	P,S,T	Tablet 200 mg<br>Tablet 400 mg<br>Powder for Injection 250 mg<br>Powder for Injection 500 mg<br>Oral liquid 400 mg/5 ml
**6.4.2–Anti Cytomegalovirus (CMV) medicines**			
6.4.2.1	Ganciclovir	S,T	Capsule 250 mg<br>Powder for Injection 500 mg
**6.4.3–Antiretroviral medicines**			
**6.4.3.1–Nucleoside reverse transcriptase inhibitors**			
	**Medicine**	**Level of Healthcare**	**Dosage form and strength**
6.4.3.1.1	Abacavir	S,T	Tablet 60 mg<br>Tablet 300 mg

Section 6.5–Antiprotozoal Medicines

6.5.1–Antiamoebic and antigiardiasis medicines

	Medicine	Level of Healthcare	Dosage form and strength
6.5.1.1	Diloxanide furoate	P,S,T	Tablet 500 mg
6.5.1.2	Metronidazole	P,S,T	Tablet 200 mg<br>Tablet 400 mg<br>Injection 500 mg/100 ml<br>Oral liquid 200 mg/5 ml

6.5.2–Antileishmaniasis medicines

	Medicine	Level of Healthcare	Dosage form and strength
6.5.2.1	Amphotericin B<br>a) Amphotericin B (conventional)<br>b) Lipid/ Liposomal Amphotericin B	S,T	Powder for Injection 50 mg
6.5.2.2	Miltefosine	P,S,T	Capsule 10 mg<br>Capsule 50 mg\
6.5.2.3	Paromomycin	P,S,T	Injection 375 mg/ml

6.5.3–Antimalarial medicines			
**6.5.3.1–For curative treatment**			
	**Medicine**	**Level of Healthcare**	**Dosage form and strength**
6.5.3.1.1	Artemether (A) + Lumefantrine (B)	P,S,T	Tablet 20 mg (A) + 120 mg (B)<br>Tablet 40 mg (A) + 240 mg (B)<br>Tablet 80 mg (A) + 480 mg (B)<br>Oral liquid 80 mg (A) + 480 mg (B)/5 ml
6.5.3.1.2	Artesunate	P,S,T	Powder for Injection 60 mg<br>Powder for Injection 120 mg
6.5.3.1.3	Artesunate (A) + Sulphadoxine - Pyrimethamine (B)	P,S,T	Combi pack (A+B)<br>1 Tablet 25 mg (A) + 1 Tablet (250 mg + 12.5 mg) (B)<br>1 Tablet 50 mg (A) + 1 Tablet (500 mg + 25 mg) (B)<br>1 Tablet 100 mg (A) + 1 Tablet (750 mg + 37.5 mg) (B)<br>1 Tablet 150 mg (A) + 2 Tablet (500 mg + 25 mg) (B)<br>1 Tablet 200 mg (A) + 2 Tablet (750 mg + 37.5 mg) (B)
6.5.3.1.4	Chloroquine	P,S,T	Tablet 150 mg<br>Oral liquid 50 mg/5 ml
6.5.3.1.5	Clindamycin	P,S,T	Capsule 150 mg<br>Capsule 300 mg

6.5.3.1.6	Primaquine	P,S,T	Tablet 2.5 mg<br>Tablet 7.5 mg<br>Tablet 15 mg
6.5.3.1.7	Quinine	P,S,T	Tablet 300 mg<br>Injection 300 mg/ml
**6.5.3.2–For prophylaxis**			
	**Medicine**	**Level of Healthcare**	**Dosage form and strength**
6.5.3.2.1	Mefloquine	T	Tablet 250 mg<br>*Only for use as chemoprophylaxis for long term travellers like military and travel troops, travelling from low endemic to high endemic area.
**6.5.4–Antipneumocystosis and antitoxoplasmosis medicines**			
	**Medicine**	**Level of Healthcare**	**Dosage form and strength**
6.5.4.1	Co-trimoxazole [Sulphamethoxazole (A) + Trimethoprim (B)]	P,S,T	Tablet 400 mg (A) + 80 mg (B)<br>Tablet 800 mg (A) + 160 mg (B)<br>Oral liquid 200 mg (A) + 40 mg (B)/5 ml
6.5.4.2	Pentamidine	S,T	Powder for Injection 200 mg

Table S4. List of anti-infective medicines added in NLEM 2022.

Medicines Added Therapeutic category wise in NLEM 2022

Section 6<br>Anti-infective medicines				
**6.1.2 -Antifilarial**				
		**Medicine**	**Level of Healthcare**	**Dosage form(s) and strength(s)**
1	6.1.5	Ivermectin	P,S,T	Tablet 6 mg<br>Tablet 12 mg
**6.2-Antibacterials**				
**6.2.1 – Beta-lactam Medicines**				
2	6.2.1.14	Meropenem	T	Powder for Injection 500 mg (as trihydrate)<br>Powder for Injection 1000 mg (as trihydrate)
**6.2.2 - Other antibacterials**				
	**Medicine**		**Level of Healthcare**	**Dosage form(s) and strength(s)**
3	6.2.2.2	Cefuroxime	P,S,T	Tablet 500 mg<br>Syrup 125 mg/ 5 mL (p)<br>Injection 1500 mg
4	6.2.2.11	Phenoxymethyl penicillin	P,S,T	Tablet 250 mg
5	6.2.2.12	Procaine Benzylpenicillin	P,S,T	Powder for injection<br>1000 mg (=1 million IU)
**6.4-Antituberculosis medicines**				

	Medicine		Level of Healthcare	Dosage form(s) and strength(s)
6	6.4.1	Amikacin	S,T	Injection 100 mg/mL<br>Injection 250 mg/mL<br>Injection 500 mg/mL
7	6.4.2	Bedaquiline	T	Tablet 100 mg
8	6.4.7	Delamanid	T	Tablet 50 mg

6.5-Antifungal medicines

	Medicine		Level of Healthcare	Dosage form(s) and strength(s)
9	6.5.5	Itraconazole	S,T	Capsule 100 mg<br>Capsule 200 mg<br>Oral liquid 10 mg/mL
10	6.5.6	Mupirocin	P,S,T	Ointment 2%
11	6.5.8	Terbinafine	P,S,T	Cream 1%

6.6.2 - Anti-Cytomegalovirus (CMV) medicines

	Medicine		Level of Healthcare	Dosage form(s) and strength(s)
12	6.6.2.1	Valganciclovir	S, T	Tablet 450 mg<br>Powder for oral solution 50 mg/mL
