# Supplementary material for: The Availability of Essential Antimicrobials in Public and Private Sector Facilities: A Cross-Sectional Survey in a District of North India
Source: Antibiotics (Basel). 2024 Jan 29;13(2):131. doi: 10.3390/antibiotics13020131 (PMC10886159; doi:10.3390/antibiotics13020131)
Supplement: Supplementary file 1 [file antibiotics-13-00131-s001.zip › antibiotics-2765598-Table S5.pdf]

DISTRICTWISE PRIMARY HEALTH CENTRES / SUB CENTRES IN Haryana			
**District**	**PHCs**		**Sub-Centres**
	**No**	**Location**	**No.**
Ambala	22	Mullana, Chaurmastpur, Shahzadpur, Brara, Bihta, Noorpur, Patrehri, Ugala, Kurali, Samlehri, Panjokhara, TharwaMajri, Ambli, Nauhani, Naggal, Boh, Saha, Kesari, Dhanana, Bhurewala, Nanyola, Chandsoli	104
Bhiwani	29	Kairu, Manheru, Jamalpur, Tosham, Loharu, Dhanana, Miran, Nakipur, Chang, Pur, Sui, Dinod, Behal, Biran, Alakhpura, Sohasara, Bamla, Jui, Lilas, Jhumpakalan, Gurera, Barwa, Kharakkalan, Talu, Dhigawa, Sandwa, Nandgaon, Dhanimahu, Jattan.	144
Charkhi Dadri	15	Gopi, Bondkalan, Jhojukalan, Maikalan, Hirodi, Manakawas, Ranila, Achina, Santokhpura, Chhapar, Kadma, Balkara, Badhra, Sanwar, Imlota,	76
Faridabad	16	Kurali, Palli Kherikalan, Tigaon, Dhouj, , PunheraKhurd, Mohna, Palla, Anangpur, , Fatehpur-billoch, Chhainsa, Fatehpur -Taga, Sikri , Dayalpur, Khabrakalan, Hijranwankalan	58
Fatehabad	24	Bhattukalan, Bhuna, Jakhal, Badopal Ratia, Jhalnia, , Birdhana, Mohamadpurrohi, Samain, Bothankalan, Bangaon, Kulan, Pirthala, Meaondkalan, Nehla, Mamupur, Pilimandori, Nagpur, Indachhui, Hassnga, Mahmara, Aharwan, Khiarati -Khera, Bighar	137
Gurugram	15	Farukhnagar, Bhorakalan, Pataudi, Ghangola, Badshahpur, Bhangrola, Mandpura, Kasan, Gurgaon-Village, Garhi-harsaru, Bhondsi, Wazirabad, , Daultabad, Nakhrola, Palra.	76
Hisar	39	Siswal, , Mangali, Aryanagar, Uklana, Mirchpur, Sisaibola, Adampur, Sorkhi, Khanda-Kheri, Ladwa, Satrodkalan, Dhansu, Pabra, Puttimangalkhan, Neolikalan, Umra, Bass, Puttisamain, Chaudhrywas, Nalwa, Agroha, Kajlan, Dobhi, Gawar, Gurana, Talwandi-Ruka, Datta, Balsamand, Kaimri, Hassangarh, Daulatupur, Chulibagrian, , Landri, Thurana, Banbhori, Bichpari, Kheri-Lohchab, Khandakheri, Sindhar.	200
Jhajjar	27	Dighal, Dhakla, Jamalpur, Dubaldhan, Badli, Chhara, Jahajgarh, Chhudani, Dujana, Birohar, Matanhail, Silani, Dubaldhan-Majra, Badsha, Mandothi, Machrauli, Bambheva, BahuJholri, Tumbaheri, Jassaurkheri, Jahangirpur, Chhuchhakwas, Salhawas, Nunamajra, Kanonda, Patauda, Bahrana.	126
Jind	33	Safidon , Jullana, Kalwa, Kharakramji, Ujhana, Kandela, Muana, Alewa, Gogrian, Shamlokalan, Rajauna-kalan, Amargarh, Deola, Dhatrath, Dhanauri, Dhamtan-Sahib, Chhatar, Ramrai, Jaijaiwanti, Nidhana, Dariyawala, Durjanpur, Dhanodakalan, Hatt, Sinsar, Sawanmal, Dumarkhan-Khurd, Naguran, Karsindhu, Karamgarh, Dahola, Uchana Kuard, Kinana.	169
Kaithal	25	Kaul, Siwan, Rajaund, Pundri, Padla, Bhagal, Dhand, Keorak, Pai, Habri, Mundri, Kangthali, Karora, Deban, Kithana, Rasina, Jakhauli, Kharkan, Batta, Balu,Teek, Arnauli, Pharal, Agondh, Sajuma.	144
Karnal	32	Taraori, Nissing, Gharaunda, Kunjpura, Ballah, Kachwa, Nigdu, , Kutail, Khukhni, Madhuban, Jundla, Barsat, Gheer, Barota, Sambhli, Gagseena, Chaura, Bhadson, Uplana, Salwan, Popra, Sagga, Jalmana, Gonder, Gudha, Ramba, Mirghan, Padha, Pardhana, Samana Bahu, Bayana, Munak.	151
Kurukshetra	22	Pehowa, Ladwa, Mathana, Jhansa, Barana, Babain, Pipli, Khanpurkohlian, Thaska-miranji, Dhurala, Siana-Saidan, Kirmach, Ismailabad, Deegh, Kalsana, Tatka, Gudha, Amin, Thol, Ramgarh-road, Barot, Sarsa	118
Nuh	22	Nuh, Firozepur-zhirka, Punhana, Ujina, Tauru, Mohammadpur-ahir, Ghasera, , Singar, Pinangwan, Tigaon, Biwan, Marora, Nagina, Padheni, Jorasi, Sikrawa, Sudaka, Jamalgarh, Bicchor, Bai, Kaliyaka, Bisru.	138
Narnaul	25	Kanina, Ateli, Nangal-sirohi, Nangal-Chaudhary , Satnali, Sehlong, Deochana, Madhogarh, Antri, Bhojawas, Sirohibihali, Bayal, Sihma, Budhwal, Balaha-kalan, Rampura, Pali, Dhanonda, Mirjapur-Bachhaud, , Mundiya- Khera, Chhilronizampur, Malrawas, Bamanwas, Mandhana, Bigopur.	120
Palwal	20	Aurangabad, Hathin, Sondhad, Alawalpur, Dadhola, Rasulpur, Tappa-bilochpur, Hasanpur, Solra, , Amarpur, Sihol, Mandkola, Uttawar, Naggaljatt, Kalsara, Bulwana, Alika, Kot, Deghot, Bhiduki.	89
Panchkula	09	Raipurrani , Pinjore, Morni, Old-Panchkula, Barwala, Hangola, Kot, Nanakpur, Surajpur	51
Panipat	20	Ahar,Dadlana Bapauli, Mathloda , Naultha, Naraina, Khotpura, Siwah,	89

		Mandi, Seenkh, Ugrakheri, Pattikalayana, Kavi, Kabri, Ujha, Chulkana, Atta, Rairkalan, Israna, Brana	
Rewari	21	Gurawara, Bawal, Nahar, Mirpur, Khol, Jatusana, Dahinajainabad, Dharuhera, Bassauda, Tankri, Fatehpuri, Bharawas, Kasola, Bawwa, Sangwari, Gudyani, Siha, Masani, Gangayacha –ahir, Bhotawas Ahir, Rathanthal	112
Rohtak	24	Kalanaur, Sampla, Kiloi, Chiri, Madina , Kahanaur, Pilana, Bahalaut, Girawar, Mokhra, Behlba, Hassangarh, Ballandh, Pakasma, Baniyani, Lakhanmajra, Sanghi, Kharwar, Samargopalpur, Ghilor-kalan, Farmana-badshpur, Karontha,Ismaila, Lakhan Majra	115
Sirsa	32	Rania, Odhan, Baraguda, Kalanwali Nathusarichopta,, Madhosinghana, Ellenabad, Juttianwali, Malekan, Goriwala, Desujodha, Ding, Panihari, Rori, Darbi, Kharia, Darba-kalan, Jagmalera, Panniwala-mota, Keharwala, Randhawa, Kaluana, Dadu, Ganga, Bani, Dhottar, Jamal**,** Bhavdeen, Bansudhar, Chautala, Kanwarpura, Kaagdana.	157
Sonipat	37	Kharkhoda, Badkhalsa, Ganaur, Juan, Mundlana, Ferozpur-banger , Purkhas, Bhainswal-kalan, Gohana, Halalpur, Bega, Farmana, Jakhauli, Dubeta, Murthal, Bidhlana, Moi-majri, Butana-zafrabad, , Rukhi, Madina, , Khanpur- kalan, Banwasa, Butana, Lath, Rohat, Bhatgaon, Mahra, Jagsi, Sisana, Sargathal Shamri, Mohana, Barodamor, Ahulana, Kundli, Rajlugarhi,Dhatoli	164
Yamuna Nagar	22	Naharpur, Saraswatinagar , Sadhaura, Chhachrauli, Bilaspur, Khizrabad, Radaur, Buria, Arnauli, Kalanaur, Sabepur, Kharwan, Kotmustraka, Muglanwali, Haibatpur, Alahar, Bhambol, Rasulpur**,** Antawa**,** Khadri, Ranjitpur, Mohri	112
**G.TOTAL**	**531**		**2650**

01.06.2020

District wise list of U-PHCs FY. 2016-17

S.No.	Name of the District	Name of Urban PHC	No. of U-PHCs
1	**Ambala**	Ravidas majri	6
		Baldev nagar	
		Durga nagar (singawala)	
		Nishan Bagh behind mill	
		Palledar mohalaa (Cant)	
		Ekta vihar (chabiyana)	
2	**Bhiwani**	M.C.Colony	3
		Dhana Ladanpur Road, Dadri gate	
		Dinod Road	
3	**Faridabad**	Nangla Enclave	13
		Shiv Durga Colony	
		Subhash Colony	
		Bharat Colony	
		Partapgarh	
		A.C. Nagar	
		Mewla	
		Saran	
		Bhim Basti Sec-18	
		Aitmadpur	
		Dabua	
		Hari Vihar	
		Adarsh Nagar	
4	**Fatehabad**	Ashok nagar	2
		Near Ratia	
		Raj nagar	1
5	**Gurgaon**	Rajiv Nagar (UrbanRCH)	18
		Laxman Vihar (UrbanRCH)	
		Firoj Gandhi Colony (UrbanRCH)	
		OM Nagar(UrbanRCH)	
		Mullahera (UrbanRCH)	
		Khandsa (UrbanRCH)	
		Rajendera Park (UrbanRCH)	
		Gandhi Nagar	

		Basai Enclave	
		Surat Nagar	
		Chaumma	
		Nathupur	
		Fazilpur	
		Naharpur Roopa	
		Sukhrali	
		Tigra	
		Patel Nagar (PPC)	
		Manesar	
**6**	**Hisar**	Patel Nagar	3
		Rishi Nagar (Ghoda farm Road)	
		Mahabir Colony	
		Char Qutub Gate	1
**7**	**Jhajjar**	Silani Gate	1
		Chhotu ram nagar	6
		Quila mohalla	
		Adarsh nagar	
		Ram nagar(barnala road)	
		Vikas nagar	
		Netaji nagar	
**8**	**Jind**	Apollo road	2
		Budha Baba Basti,Bhiwani road	
**9**	**Kaithal**	Shakti Nagar	3
		GH OLD Hospital	
		Near ITI, Jind Road	
**10**	**Karnal**	Dhobi Mohalla	4
		Indira Colony	
		Ram Nagar	
		Shiv Colony	
**11**	**Kurukshetra**	Mohan nagar	3
		Krishna nagar gamri	
		Majri mohallah	
**12**	**Palwal**	Kasba Mohalla	2
		Sallagarh	

**13**	**Panchkula**	Bhero ki Sher Kalka	1
**14**	**Panipat**	Peri Urban Ugrakheri	6
		Raj Nagar	
		Rajeev Colony	
		Batra Colony	
		Khatik Basti	
		Hari Singh Colony (peri urban kabri)	
**15**	**Rewari**	Qutubpur	3
		Rajeev Nagar	
		Daruheda	
**16**	**Rohtak**	Gau Karan(roop nagar nehru colony)	4
		Rajendra Colony(Hari singh colony)	
		Ekta Colony	
		Shukhpura Chownk	
**17**	**Sirsa**	Chattatgarh patti	4
		Khairpur	
		Inderpuri mohalla (JJ colony, Harijan basti + Rani chungi peer basti)	
		Ther mohalla	
		PHC Dabwali	1
**18**	**Sonepat**	Subhash Nagar	3
		Kailash Colony (Patel nagar)	
		Jatwara (Jamalpura)	
**19**	**Yamunanagar**	Azad nagar	95
		Gandhi nagar	
		Ganganagar	
		Mukherjee Park	
		Old Hamida	
Total			95
Note: Two FRUs in Distt Faridabad			

DISTRICTWISE COMMUNITY HEALTH CENTRES		
**District**	**CHCs**	
	**No**	**Location**
Ambala	5	Mullana, Chaurmastpur, Shahzadpur, Brara, Ambli
Bhiwani	7	Kairu, Manheru, Jamalpur, Tosham,<br>**50 Bedded CHC** Loharu<br>**Block PHC** Dhanana, Miran
Charkhi Dadri	3	Gopi, Bondkalan, Jhojukalan,
Faridabad	4	Kurali Pali, Tigaon<br>**Block PHC** Kheri Kalan
Fatehabad	6	Bhattukalan, Bhuna, Jakhal Badopal, Bothankalan<br>**CHC cum 50 Bedded Hospital** Ratia
Gurugram	4	Farukhnagar, Bhora Kalan<br>**50 Bedded CHC** Pataudi<br>**Block PHC** Ghangola
Hisar	9	Siswal, Mangali, Aryanagar Uklanan Mirchpur, SisaiBola, Khanda Kheri<br>**CHC cum 50 Bedded Hospital** Adampur<br>**Block PHC** Sorkhi
Jhajjar	6	Dighal, Dhakla, Jamalpur, Dubaldhan, Badli<br>**Block PHC** Charra
Jind	**8**	Safidon, Jullana, Kalwa, Kharakramji, Ujhana, Kandela, Muana Alewa,
Kaithal	4	Guhla, Kaul, Siwan, Rajaund, Pundri
Karnal	5	Taraori, Nissing, Gharaunda,, Kunjpura<br>**Block PHC** Ballah
Kurukshetra	6	Pehowa, Ladwa, Mathana, Jhansa, Barana, Babain
Nuh	4	Nuh, Ferozepur Zhirka, Punhana, Tauru
Narnaul	7	Kanina,, Ateli, Nangal-sirohi_, Nangal-chaudhry, Satnali, Sehlang<br>**Functional** Dochana
Palwal	5	Aurangabad, Hathin Sondhad, Alawalpur<br>**Block PHC** Dudhola
Panchkula	2	Raipurrani, Nanakpur
Panipat	7	Ahar Dadlana, Bapoli, Mathloda , Naultha ,Naraina, khotpura
Rewari	5	Gurawara, Bawal, Nahar, Mirpur, Khol
Rohtak	7	Kahnaur, Sampla, Kiloi, Chiri, Madina<br>**50 Bedded CHC** Kalanaur Lakhan Majra
Sirsa	8	Rania, Odhan , Bara-Gudda Kalanwali, Nathusari-Chopta , Madhosinghana<br>**CHC cum 50 Bedded Hospital** Ellenabad Chautala
Sonipat	9	Kharkhauda, Badkhalsa, Ganaur, , Juan, Mundlana Firozepur -bangar Purkhas Bhainswal -kalan<br>**50 Bedded CHC** Gohana
Yamuna Nagar	7	Naharpur, Saraswatinagar , Sadhaura,Chhachhrauli Bilaspur<br>**Block PHC** Khizrabad<br>**50 Bedded CHC** Radaur
**G.TOTAL**	**128**	

01.06.2020

Medicine India (//www.medicineindia.org)
/ Pharmacists,Chemists,Drugstores In India (//www.medicineindia.org/pharmacies-chemists-drugstores-in-india)
/ Rohtak (//www.medicineindia.org/pharmacies-chemists-drugstores-in-city/953/rohtak)

+ Add New Pharmacy (//www.medicineindia.org/pharmacies-chemists-drugstores-in-india/add-new-pharmacy)

Pharmacies, Chemists & Drugstores in Rohtak

Filter Pharmacies:

Pharmacy/Chemist/Drugstore Name
Aasta Medical Store (//www.medicineindia.org/pharmacy-chemist-drugstore-details/79852/aasta-medical-store)
Aggarwal Medical Store (//www.medicineindia.org/pharmacy-chemist-drugstore-details/79863/aggarwal-medical-store)
Aggarwal Medicla Store (//www.medicineindia.org/pharmacy-chemist-drugstore-details/79915/aggarwal-medicla-store)
Aghi Pharmacy (//www.medicineindia.org/pharmacy-chemist-drugstore-details/79891/aghi-pharmacy)
Ajit Medical Agencies (//www.medicineindia.org/pharmacy-chemist-drugstore-details/79935/ajit-medical-agencies)
Anand Medicos (//www.medicineindia.org/pharmacy-chemist-drugstore-details/79820/anand-medicos)
Anil Medicos Pvt Ltd (//www.medicineindia.org/pharmacy-chemist-drugstore-details/79827/anil-medicos-pvt-ltd)
Arun Medicos (//www.medicineindia.org/pharmacy-chemist-drugstore-details/79894/arun-medicos)
Aryan Medical Hall (//www.medicineindia.org/pharmacy-chemist-drugstore-details/79897/aryan-medical-hall)
Aryan Pharma (//www.medicineindia.org/pharmacy-chemist-drugstore-details/82654/aryan-pharma)
Asho Madicoes (//www.medicineindia.org/pharmacy-chemist-drugstore-details/79832/asho-madicoes)
Ashok A Medical Hall (//www.medicineindia.org/pharmacy-chemist-drugstore-details/79953/ashok-a-medical-hall)
Ayusearch Drugs & Lab (//www.medicineindia.org/pharmacy-chemist-drugstore-details/79947/ayusearch-drugs-lab)
B D Pharma (//www.medicineindia.org/pharmacy-chemist-drugstore-details/79826/b-d-pharma)
Bhagwati Medical Agencies (//www.medicineindia.org/pharmacy-chemist-drugstore-details/79922/bhagwati-medical-ag
Bharat Medical Company (//www.medicineindia.org/pharmacy-chemist-drugstore-details/79813/bharat-medical-compar
Bhatia Medical Hall (//www.medicineindia.org/pharmacy-chemist-drugstore-details/79875/bhatia-medical-hall)
Bhatia Medicos (//www.medicineindia.org/pharmacy-chemist-drugstore-details/79909/bhatia-medicos)
Bhutani Medical Hall (//www.medicineindia.org/pharmacy-chemist-drugstore-details/79828/bhutani-medical-hall)

Bhutani Medical Hall (//www.medicineindia.org/pharmacy-chemist-drugstore-details/79866/bhutani-medical-hall)
Bhutani Medical Store (//www.medicineindia.org/pharmacy-chemist-drugstore-details/79929/bhutani-medical-store)
Bramh Aushdhalay (//www.medicineindia.org/pharmacy-chemist-drugstore-details/79837/bramh-aushdhalay)
Chahal Medical Hall (//www.medicineindia.org/pharmacy-chemist-drugstore-details/82647/chahal-medical-hall)
Chaman Medicos (//www.medicineindia.org/pharmacy-chemist-drugstore-details/79938/chaman-medicos)
Chawla Medical Hall (//www.medicineindia.org/pharmacy-chemist-drugstore-details/79869/chawla-medical-hall)
Chawla Medicos (//www.medicineindia.org/pharmacy-chemist-drugstore-details/79855/chawla-medicos)
Chirag Medicose (//www.medicineindia.org/pharmacy-chemist-drugstore-details/79946/chirag-medicose)
Chugh Medicos (//www.medicineindia.org/pharmacy-chemist-drugstore-details/79818/chugh-medicos)
City Medicos (//www.medicineindia.org/pharmacy-chemist-drugstore-details/79936/city-medicos)
D.E Medicose (//www.medicineindia.org/pharmacy-chemist-drugstore-details/79835/de-medicose)
DEV Pharma (//www.medicineindia.org/pharmacy-chemist-drugstore-details/79839/dev-pharma)
Dahiya Medical Store (//www.medicineindia.org/pharmacy-chemist-drugstore-details/79850/dahiya-medical-store)
Daya Nand Medicos Agencies (//www.medicineindia.org/pharmacy-chemist-drugstore-details/79908/daya-nand-medico
Deep Madical Hall (//www.medicineindia.org/pharmacy-chemist-drugstore-details/79944/deep-madical-hall)
Deep Medicos (//www.medicineindia.org/pharmacy-chemist-drugstore-details/79913/deep-medicos)
Dev Pharma (//www.medicineindia.org/pharmacy-chemist-drugstore-details/82461/dev-pharma)
Dhall Medicose (//www.medicineindia.org/pharmacy-chemist-drugstore-details/79876/dhall-medicose)
Dhruv Medical Hall (//www.medicineindia.org/pharmacy-chemist-drugstore-details/79889/dhruv-medical-hall)
Dhruv Medical Store (//www.medicineindia.org/pharmacy-chemist-drugstore-details/79882/dhruv-medical-store)
Dinesh Medicos (//www.medicineindia.org/pharmacy-chemist-drugstore-details/79847/dinesh-medicos)
Doctor Medicos (//www.medicineindia.org/pharmacy-chemist-drugstore-details/79848/doctor-medicos)
Dr Jains Life Science (//www.medicineindia.org/pharmacy-chemist-drugstore-details/79887/dr-jains-life-science)
Durga Medical Hall (//www.medicineindia.org/pharmacy-chemist-drugstore-details/79883/durga-medical-hall)
Friends Pharma (//www.medicineindia.org/pharmacy-chemist-drugstore-details/79815/friends-pharma)
G K Medicos (//www.medicineindia.org/pharmacy-chemist-drugstore-details/79945/g-k-medicos)

Gandhi Medical Hall (//www.medicineindia.org/pharmacy-chemist-drugstore-details/79921/gandhi-medical-hall)
Gaurav Medicos (//www.medicineindia.org/pharmacy-chemist-drugstore-details/79910/gaurav-medicos)
Giriraj Medicos (//www.medicineindia.org/pharmacy-chemist-drugstore-details/79900/giriraj-medicos)
Giriraj Medicos (//www.medicineindia.org/pharmacy-chemist-drugstore-details/79911/giriraj-medicos)
Godara Medical Hall (//www.medicineindia.org/pharmacy-chemist-drugstore-details/79934/godara-medical-hall)
Goyal Medical Agency (//www.medicineindia.org/pharmacy-chemist-drugstore-details/79838/goyal-medical-agency)
Goyal Medicall Hall (//www.medicineindia.org/pharmacy-chemist-drugstore-details/79952/goyal-medicall-hall)
Gulati Medical Hall Gulati Madi (//www.medicineindia.org/pharmacy-chemist-drugstore-details/79830/gulati-medical-hal
Gulati Medicose (//www.medicineindia.org/pharmacy-chemist-drugstore-details/79877/gulati-medicose)
Gunjan Medical Hall (//www.medicineindia.org/pharmacy-chemist-drugstore-details/79833/gunjan-medical-hall)
Gunjan Medical Hall (//www.medicineindia.org/pharmacy-chemist-drugstore-details/79874/gunjan-medical-hall)
Gupta Medicos (//www.medicineindia.org/pharmacy-chemist-drugstore-details/79819/gupta-medicos)
Hare Krishana Medicos (//www.medicineindia.org/pharmacy-chemist-drugstore-details/79902/hare-krishana-medicos)
Haryana Medical Hall (//www.medicineindia.org/pharmacy-chemist-drugstore-details/79933/haryana-medical-hall)
Hemant Medical Agencies (//www.medicineindia.org/pharmacy-chemist-drugstore-details/79823/hemant-medical-agen
Huria Medical Store (//www.medicineindia.org/pharmacy-chemist-drugstore-details/79896/huria-medical-store)
Huria Medicose (//www.medicineindia.org/pharmacy-chemist-drugstore-details/79872/huria-medicose)
J P Medical Agencies (//www.medicineindia.org/pharmacy-chemist-drugstore-details/79926/j-p-medical-agencies)
JAI Durga Medical Hall (//www.medicineindia.org/pharmacy-chemist-drugstore-details/79919/jai-durga-medical-hall)
JAI Hanuman Medicals (//www.medicineindia.org/pharmacy-chemist-drugstore-details/79939/jai-hanuman-medicals)
JAI Shri Ram Medicos (//www.medicineindia.org/pharmacy-chemist-drugstore-details/79829/jai-shri-ram-medicos)
Jagdamba Medical Store (//www.medicineindia.org/pharmacy-chemist-drugstore-details/79903/jagdamba-medical-stor
Jagdambe Medicos (//www.medicineindia.org/pharmacy-chemist-drugstore-details/79845/jagdambe-medicos)
Janta Medical Store (//www.medicineindia.org/pharmacy-chemist-drugstore-details/79940/janta-medical-store)
Jeet Medicos (//www.medicineindia.org/pharmacy-chemist-drugstore-details/79941/jeet-medicos)
Jenta Medical Store (//www.medicineindia.org/pharmacy-chemist-drugstore-details/79858/jenta-medical-store)

Jia Shri Ram Medicose (//www.medicineindia.org/pharmacy-chemist-drugstore-details/79949/jia-shri-ram-medicose)
Juneja Medical Store (//www.medicineindia.org/pharmacy-chemist-drugstore-details/79870/juneja-medical-store)
Kamal Medical Hall (//www.medicineindia.org/pharmacy-chemist-drugstore-details/79948/kamal-medical-hall)
Kamal Medical Store (//www.medicineindia.org/pharmacy-chemist-drugstore-details/79950/kamal-medical-store)
Kataria Medical Hall (//www.medicineindia.org/pharmacy-chemist-drugstore-details/79812/kataria-medical-hall)
Kathuria Dawai Wala (//www.medicineindia.org/pharmacy-chemist-drugstore-details/79854/kathuria-dawai-wala)
Khurana Dawakhana (//www.medicineindia.org/pharmacy-chemist-drugstore-details/79840/khurana-dawakhana)
Khurana Pharmacy (//www.medicineindia.org/pharmacy-chemist-drugstore-details/79816/khurana-pharmacy)
Kissan Medical Agency (//www.medicineindia.org/pharmacy-chemist-drugstore-details/79831/kissan-medical-agency)
Krishana Medicose (//www.medicineindia.org/pharmacy-chemist-drugstore-details/79954/krishana-medicose)
Kurana Farmashi (//www.medicineindia.org/pharmacy-chemist-drugstore-details/79884/kurana-farmashi)
M.J. Sales Chemist (//www.medicineindia.org/pharmacy-chemist-drugstore-details/79878/mj-sales-chemist)
Malik Medical Agencies (//www.medicineindia.org/pharmacy-chemist-drugstore-details/79906/malik-medical-agencies)
Malik Medical Store (//www.medicineindia.org/pharmacy-chemist-drugstore-details/79871/malik-medical-store)
Malik Medical Store (//www.medicineindia.org/pharmacy-chemist-drugstore-details/79898/malik-medical-store)
Midha Medical Store (//www.medicineindia.org/pharmacy-chemist-drugstore-details/79873/midha-medical-store)
Monu medical hall (//www.medicineindia.org/pharmacy-chemist-drugstore-details/81766/monu-medical-hall)
Nagpal Medical Store (//www.medicineindia.org/pharmacy-chemist-drugstore-details/79824/nagpal-medical-store)
National Medical Agencies (//www.medicineindia.org/pharmacy-chemist-drugstore-details/79927/national-medical-agen
National Medical Hall (//www.medicineindia.org/pharmacy-chemist-drugstore-details/79925/national-medical-hall)
New Jyoti Medical Hall (//www.medicineindia.org/pharmacy-chemist-drugstore-details/79864/new-jyoti-medical-hall)
Niranjan Dass Jaswant RAI (//www.medicineindia.org/pharmacy-chemist-drugstore-details/79892/niranjan-dass-jaswar
Nishil Medicos (//www.medicineindia.org/pharmacy-chemist-drugstore-details/82250/nishil-medicos)
Nishil Medicos (//www.medicineindia.org/pharmacy-chemist-drugstore-details/82697/nishil-medicos)
Nitin Medical Store (//www.medicineindia.org/pharmacy-chemist-drugstore-details/79867/nitin-medical-store)
OM Medical Agency (//www.medicineindia.org/pharmacy-chemist-drugstore-details/79811/om-medical-agency)

OM Medicos (//www.medicineindia.org/pharmacy-chemist-drugstore-details/79901/om-medicos)
Om Medical Hall (//www.medicineindia.org/pharmacy-chemist-drugstore-details/79809/om-medical-hall)
Pal Medical Hall (//www.medicineindia.org/pharmacy-chemist-drugstore-details/79856/pal-medical-hall)
Pambu ENT (//www.medicineindia.org/pharmacy-chemist-drugstore-details/79942/pambu-ent)
Paras Medicals (//www.medicineindia.org/pharmacy-chemist-drugstore-details/79844/paras-medicals)
Paras Medicos (//www.medicineindia.org/pharmacy-chemist-drugstore-details/79886/paras-medicos)
Parkash Medicos (//www.medicineindia.org/pharmacy-chemist-drugstore-details/79907/parkash-medicos)
Pavit Medical Agencies (//www.medicineindia.org/pharmacy-chemist-drugstore-details/79821/pavit-medical-agencies)
Pawaia Medicals (//www.medicineindia.org/pharmacy-chemist-drugstore-details/79836/pawaia-medicals)
Pawan Medical Hall (//www.medicineindia.org/pharmacy-chemist-drugstore-details/79930/pawan-medical-hall)
Pharma Ghar (//www.medicineindia.org/pharmacy-chemist-drugstore-details/82528/pharma-ghar)
Prakash Medical Store (//www.medicineindia.org/pharmacy-chemist-drugstore-details/79841/prakash-medical-store)
Prakash Medicos (//www.medicineindia.org/pharmacy-chemist-drugstore-details/79814/prakash-medicos)
Prem Medicose (//www.medicineindia.org/pharmacy-chemist-drugstore-details/79868/prem-medicose)
Punjab Medical & General Store (//www.medicineindia.org/pharmacy-chemist-drugstore-details/79817/punjab-medical-
Punjab Medical Genral Store (//www.medicineindia.org/pharmacy-chemist-drugstore-details/79843/punjab-medical-gen
R K Medical Hall (//www.medicineindia.org/pharmacy-chemist-drugstore-details/79923/r-k-medical-hall)
R P Medical Store (//www.medicineindia.org/pharmacy-chemist-drugstore-details/79905/r-p-medical-store)
Rachit Medical Hall (//www.medicineindia.org/pharmacy-chemist-drugstore-details/79943/rachit-medical-hall)
Radha Swami Medical Hall (//www.medicineindia.org/pharmacy-chemist-drugstore-details/79822/radha-swami-medical
Rahul Medical Hall (//www.medicineindia.org/pharmacy-chemist-drugstore-details/79862/rahul-medical-hall)
Raja Medicos (//www.medicineindia.org/pharmacy-chemist-drugstore-details/79890/raja-medicos)
Raja Medicos (//www.medicineindia.org/pharmacy-chemist-drugstore-details/79912/raja-medicos)
Rajiv Medical Store (//www.medicineindia.org/pharmacy-chemist-drugstore-details/79834/rajiv-medical-store)
Ranjandas Jaswant RAI (//www.medicineindia.org/pharmacy-chemist-drugstore-details/79937/ranjandas-jaswant-rai)

Riddhi shree Medicos (//www.medicineindia.org/pharmacy-chemist-drugstore-details/79893/riddhi-shree-medicos)
Rohilla Medicos (//www.medicineindia.org/pharmacy-chemist-drugstore-details/79849/rohilla-medicos)
Rohtak Medical Agencies (//www.medicineindia.org/pharmacy-chemist-drugstore-details/79808/rohtak-medical-agencie
SH. SAI Baba Medicos (//www.medicineindia.org/pharmacy-chemist-drugstore-details/79810/sh-sai-baba-medicos)
Sabharwal Medical Store (//www.medicineindia.org/pharmacy-chemist-drugstore-details/79920/sabharwal-medical-stor
Sachdeva Medical Agencies (//www.medicineindia.org/pharmacy-chemist-drugstore-details/79928/sachdeva-medical-a
Sanjay Chemist (//www.medicineindia.org/pharmacy-chemist-drugstore-details/79842/sanjay-chemist)
Sanjeev Ayurvedic Store (//www.medicineindia.org/pharmacy-chemist-drugstore-details/82388/sanjeev-ayurvedic-store
Sawan Medicos (//www.medicineindia.org/pharmacy-chemist-drugstore-details/79895/sawan-medicos)
Shanti Medicos (//www.medicineindia.org/pharmacy-chemist-drugstore-details/79904/shanti-medicos)
Shirdi Medicos (//www.medicineindia.org/pharmacy-chemist-drugstore-details/79888/shirdi-medicos)
Shiv Medical Agencies (//www.medicineindia.org/pharmacy-chemist-drugstore-details/79932/shiv-medical-agencies)
Shiv Medical Store (//www.medicineindia.org/pharmacy-chemist-drugstore-details/79853/shiv-medical-store)
Shiv Pharma Traders (//www.medicineindia.org/pharmacy-chemist-drugstore-details/79825/shiv-pharma-traders)
Shiv Shakti Medical Store (//www.medicineindia.org/pharmacy-chemist-drugstore-details/79859/shiv-shakti-medical-stc
Shree Ganpati Medicose (//www.medicineindia.org/pharmacy-chemist-drugstore-details/79879/shree-ganpati-medicose
Shree hari medical hall (//www.medicineindia.org/pharmacy-chemist-drugstore-details/82656/shree-hari-medical-hall)
Singhle Medical Store (//www.medicineindia.org/pharmacy-chemist-drugstore-details/79881/singhle-medical-store)
Subhash Medical Hall (//www.medicineindia.org/pharmacy-chemist-drugstore-details/79931/subhash-medical-hall)
Subhash Medicos (//www.medicineindia.org/pharmacy-chemist-drugstore-details/79846/subhash-medicos)
Suneja Medical (//www.medicineindia.org/pharmacy-chemist-drugstore-details/79924/suneja-medical)
Sunny Medical Store (//www.medicineindia.org/pharmacy-chemist-drugstore-details/79861/sunny-medical-store)
Supreme Medical (//www.medicineindia.org/pharmacy-chemist-drugstore-details/79918/supreme-medical)
Taneja Medical Store (//www.medicineindia.org/pharmacy-chemist-drugstore-details/79851/taneja-medical-store)
Thukral Medical Hall (//www.medicineindia.org/pharmacy-chemist-drugstore-details/79880/thukral-medical-hall)

Tushar Medicine Point (//www.medicineindia.org/pharmacy-chemist-drugstore-details/79899/tushar-medicine-point)

V K Medicos (//www.medicineindia.org/pharmacy-chemist-drugstore-details/79914/v-k-medicos)

Vanshika Trading Co (//www.medicineindia.org/pharmacy-chemist-drugstore-details/79885/vanshika-trading-co)
Vijay Medical Store (//www.medicineindia.org/pharmacy-chemist-drugstore-details/79917/vijay-medical-store)
Vinit Medicose (//www.medicineindia.org/pharmacy-chemist-drugstore-details/79865/vinit-medicose)
Vishnu Medicals (//www.medicineindia.org/pharmacy-chemist-drugstore-details/79916/vishnu-medicals)
Yash Medicos (//www.medicineindia.org/pharmacy-chemist-drugstore-details/79857/yash-medicos)
Yash Medicos (//www.medicineindia.org/pharmacy-chemist-drugstore-details/79860/yash-medicos)
Yetichem Labs Pvt Ltd (//www.medicineindia.org/pharmacy-chemist-drugstore-details/79951/yetichem-labs-pvt-ltd)

Recently Added Pharmacies

NASA PHARMA, Raebareli (//www.medicineindia.org/pharmacy-chemist-drugstore-details/82746/nasa-pharma)

Rajasthan medical store, Ajmer (//www.medicineindia.org/pharmacy-chemist-drugstore-details/82745/rajasthan-medical-store)

Manko pharmacy, Mumbai (//www.medicineindia.org/pharmacy-chemist-drugstore-details/82744/manko-pharmacy)

GuruNanak Medical Agency, Panipat (//www.medicineindia.org/pharmacy-chemist-drugstore-details/82743/gurunanak-medical-agency)

VARDHMAN MEDICALHALL, Nalanda (//www.medicineindia.org/pharmacy-chemist-drugstore-details/82742/vardhman-medicalhall)

Modern Health Care, Srinagar (//www.medicineindia.org/pharmacy-chemist-drugstore-details/82741/modern-health-care)

a.s medicles, Aurangabad (//www.medicineindia.org/pharmacy-chemist-drugstore-details/82740/as-medicles)

MAHALAXMI MEDICO, Pune (//www.medicineindia.org/pharmacy-chemist-drugstore-details/82739/mahalaxmi-medico)

Life Care Medical, Jorhat (//www.medicineindia.org/pharmacy-chemist-drugstore-details/82738/life-care-medical)

Hemkunt medical hall, Moradabad (//www.medicineindia.org/pharmacy-chemist-drugstore-details/82737/hemkunt-medical-hall)

Important Links:

Ministry of Health & Family Welfare-Government of India (http://mohfw.nic.in)

Department of Health Research (DHR), Government of India (http://www.dhr.gov.in)

Department of Indian Systems of Medicine and Homoeopathy (http://indianmedicine.nic.in)

Pharmacopoeial Laboratory for Indian Medicine (PLIM) (http://www.plimism.nic.in)

Medicine Information Centre (http://www.niper.gov.in/medicine.htm)
